# Idiopathic Orbital Inflammatory Disease in a Pediatric Patient: A Case Report Highlighting the Diagnostic Reasoning and Treatment Strategy

**DOI:** 10.7759/cureus.67569

**Published:** 2024-08-23

**Authors:** Ahmed Elashmawy, Catrina Stephan, Arhan Shetty, Ashley Yangouyian, Saima Sharif

**Affiliations:** 1 Pediatrics, Wayne State University School of Medicine, Detroit, USA; 2 Pediatrics, Michigan State University College of Human Medicine, Lansing, USA; 3 Neonatology, Central Michigan University College of Medicine, Detroit, USA

**Keywords:** nonspecific orbital inflammation, diagnosis of exclusion, multidisciplinary management, ocular proptosis, idiopathic orbital inflammatory disease

## Abstract

Idiopathic orbital inflammatory disease (IOID) is a rare and poorly understood condition characterized by inflammation of the orbital tissues without an identifiable cause. This disorder can lead to symptoms such as proptosis, pain, and visual disturbances. We present the case of an eight-year-old female diagnosed with IOID who was admitted to the hospital with worsening right eye redness, proptosis, and pain. Her clinical course included significant right eye lagophthalmos, exposure keratopathy, and a corneal ulcer. Management involved a multidisciplinary approach with consultations from ophthalmology and rheumatology, treatment with corticosteroids, and supportive care. This case underscores the importance of early recognition and a comprehensive management strategy to improve outcomes for patients with IOID.

## Introduction

Idiopathic orbital inflammatory disease (IOID), also referred to as orbital pseudotumor or nonspecific orbital inflammation, is a benign yet severe inflammatory condition confined mostly to the orbit. The resulting inflammation can affect various orbital structures, leading to a wide range of clinical presentations. Idiopathic orbital inflammatory disease can manifest in different forms depending on the affected tissues, such as anterior, diffuse, posterior, or myositis types, with dacryoadenitis being the most common presentation, affecting about 50% of cases [1]. The lacrimal gland is most frequently involved (32%), followed closely by the extraocular muscles (superior rectus, inferior rectus, medial rectus, lateral rectus, superior oblique, and inferior oblique) at 29% [2]. Idiopathic orbital inflammatory disease accounts for approximately 8%-10% of all orbital mass lesions, making it a significant differential diagnosis in patients presenting with orbital masses [2].

The pathogenesis of IOID remains unclear, but it is believed to involve immunological pathways, possibly linked to underlying autoimmune disorders such as systemic lupus erythematosus, inflammatory bowel disease, rheumatoid arthritis, thyroid disease, sarcoidosis, myasthenia gravis, and various vasculitis autoimmune diseases, including granulomatosis with polyangiitis [3]. This class of disorders may predispose patients to an exaggerated inflammatory response within the orbital tissues, leading to the development of IOID. The wide-ranging differential diagnoses for IOID, including infections, neoplasms, and systemic inflammatory disorders, make diagnosis and management particularly challenging [4]. The clinical presentation of IOID is variable, but common symptoms include proptosis, pain, periorbital swelling, and diplopia [5]. Notably, IOID is often mistaken for orbital cellulitis and pre-septal cellulitis, both of which present with overlapping features such as swelling, erythema, and pain [6].

Imaging modalities such as MRI and CT scans are crucial for assessing the extent of orbital involvement and excluding other potential causes of the symptoms. Histopathological examination may be necessary in certain cases to confirm the diagnosis and rule out malignancies. Once diagnosed, the mainstay of treatment for IOID is systemic corticosteroids, which are highly effective in reducing inflammation and alleviating symptoms [7]. The typical course involves a high-dose corticosteroid regimen followed by a gradual taper over several weeks to months, depending on the patient's response. In cases where corticosteroids are insufficient or contraindicated, additional immunosuppressive therapies such as methotrexate, azathioprine, or cyclosporine may be used [3]. All in all, diagnosing IOID requires a comprehensive clinical evaluation, laboratory testing, and imaging studies to rule out other conditions. Refractory cases or those with structural complications may require surgical intervention, including orbital decompression or biopsy for further evaluation [2]. Early and aggressive treatment is essential to prevent complications such as optic neuropathy, permanent vision loss, and orbital fibrosis, which can result from prolonged inflammation [2]. This case report presents the clinical presentation, diagnostic workup, and management of an eight-year-old female diagnosed with IOID, highlighting the importance of a thorough diagnostic approach and the complexities of treating this condition in the pediatric population.

## Case presentation

**Figure 1:**
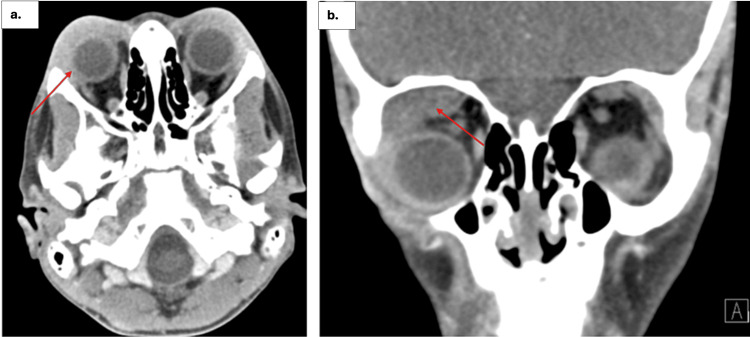
A CT scan of the orbit/sella/ear (with contrast) taken 18 months prior a. The axial CT scan revealed asymmetric proptosis, with greater enlargement of the lacrimal gland on the right side compared to the left. The enlarged right lacrimal gland is indicated by a red arrow. b. The coronal CT scan also demonstrated the enlargement of the right lacrimal gland. In this view, the enlarged gland is similarly pointed out with a red arrow. In both the axial and coronal planes, the red arrows highlight the enlargement of the right lacrimal gland, with no evidence of an abscess.

An eight-year-old female with a history of bilateral lacrimal gland enlargement, status post partial excision, presented to the ED with an acute onset of worsening right eye redness, proptosis, and pain with eye movements, all developing within a week. The symptoms began suddenly with mild discomfort and swelling on the first day, followed by noticeable redness and mild proptosis by the second day. By the fourth day, the pain had intensified, and by the sixth day, the severity had escalated to pronounced right eye redness, increased proptosis, and significant difficulty in closing the right eye, prompting her mother to bring her to the ED. She reported photophobia but maintained intact vision without blurriness or floaters. A biopsy taken 18 months prior had revealed lacrimal gland inflammation, leading to a diagnosis of nonspecific orbital inflammatory syndrome and guiding the decision for partial excision, which was performed to obtain tissue for histopathological analysis and to relieve the acute symptomatic proptosis. Previous imaging taken at the same time had shown bilateral proptosis with downward deviation, likely due to asymmetrically enlarged bilateral lacrimal glands (Figure 1). Given her medical history and the rapid progression of symptoms, it was likely that the patient was experiencing an acute exacerbation of a chronic condition. Prior workups for autoimmune and neoplastic processes had been negative. The patient also had no history of fevers, weight loss, night sweats, recent trauma, or upper respiratory infection symptoms.

In the ED, an ophthalmologist evaluated her and immediately initiated treatment with IV methylprednisone (1.5 mg/kg) due to the acute and rapidly progressing nature of her symptoms, which included significant proptosis, pain, and risk of further complications like vision loss. Given the urgency of reducing inflammation to prevent potential damage to the optic nerve and other ocular structures, the decision was made to administer steroids before completing lab work. This approach was chosen to promptly manage the severe inflammatory response while lab work was concurrently obtained to identify any underlying infectious or autoimmune processes. Following the steroid administration, artificial tears were applied every two hours in both eyes. Additionally, to facilitate a thorough examination of the fundus and rule out any posterior segment pathology, her eyes were dilated using both 2.5% phenylephrine and 1% tropicamide drops. The use of 2.5% phenylephrine, a sympathomimetic agent, was intended to achieve mydriasis by stimulating the dilator muscle of the iris, while the 1% tropicamide, an anticholinergic agent, was used to inhibit the sphincter muscle, leading to more effective and sustained dilation. This combination was chosen to ensure maximal pupillary dilation, given the need to carefully examine the posterior segment of the eye in a patient presenting with proptosis and potential optic nerve involvement. The laboratory evaluation revealed an elevated C-reactive protein (CRP), slightly elevated white blood cell (WBC) count, and elevated platelet count, all suggestive of an inflammatory process with a possible infectious component. Thyroid studies, lactate dehydrogenase (LDH), and uric acid levels were within normal ranges. The exact values and reference ranges for these results are detailed in Table 1.

**Table 1:** Summary of emergency room laboratory findings The elevated CRP of 15 mg/L suggests inflammation, while the slightly elevated WBC of 11.4 × 10^9/L indicates a possible ongoing infection or inflammatory response. These findings collectively suggested an inflammatory process with a potential infectious etiology, requiring further clinical assessment and correlation with the patient’s symptoms and medical history. Units: mg/L: milligrams per liter; × 10^9/L: billion cells per liter; µIU/mL: micro-international units per milliliter; ng/mL: nanograms per milliliter; ng/dL: nanograms per deciliter; U/L: Units per liter; mg/dL: milligrams per deciliter

Lab Test	Result	Reference range	Interpretation
C-reactive protein (CRP)	15 mg/L	< 3 mg/L	Elevated, suggests inflammation
White blood cell (WBC) count	11.4 × 10^9/L	4.0-10.0 × 10^9/L	Slightly elevated, possible infection/inflammatory response
Platelets	492 × 10^9/L	150-400 × 10^9/L	Elevated
Thyroid-stimulating hormone (TSH)	2.5 µIU/mL	0.4-4.0 µIU/mL	Within normal range
Triiodothyronine (T3)	1.2 ng/mL	0.8-2.0 ng/mL	Within normal range
Thyroxine (T4)	1.0 ng/dL	0.8-1.8 ng/dL	Within normal range
Lactate dehydrogenase (LDH)	150 U/L	120-250 U/L	Within normal range
Uric acid	4.5 mg/dL	3.5-7.2 mg/dL	Within normal range

**Figure 2:**
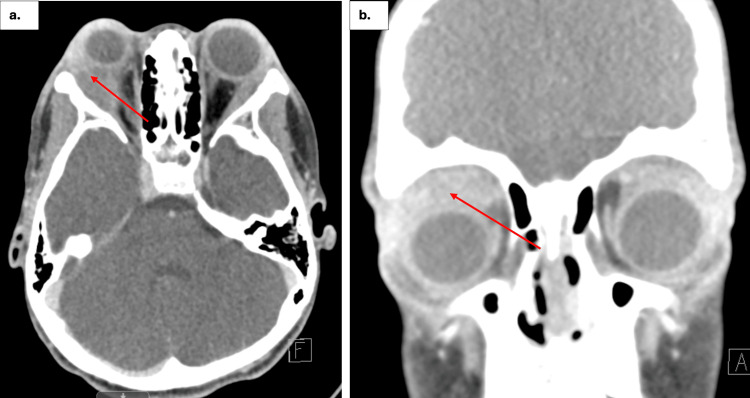
Latest CT scan of the orbit/sella/ear (with contrast) a. The axial CT scan (left) shows asymmetric proptosis with greater enlargement of the right lacrimal gland compared to the left. The right lacrimal gland appears larger compared to previous imaging (Figure 1), resulting in an increased mass effect on the right globe. The enlarged right lacrimal gland is indicated by a red arrow. b. The coronal CT scan (right) also demonstrates the enlargement of the right lacrimal gland. In this view, the enlarged gland is highlighted with a red arrow. In both the axial and coronal planes, the red arrows point out the enlarged right lacrimal gland, with no evidence of an abscess.

A recent CT scan of the orbit revealed asymmetric proptosis (right greater than left as shown in Figure 2) related to asymmetrically enlarged lacrimal glands, with the right slightly larger than previously imaged. No abscess was present. An ophthalmologist later noted corneal ulceration in the right eye, measuring approximately 2 mm in diameter, located centrally on the cornea, likely due to severe lagophthalmos and exposure. Examination also revealed decreased visual acuity in the right eye, reduced to 20/60 in the right eye, with significant conjunctival injection and stromal thinning at the site of the ulcer. Despite the presence of keratitis and the corneal ulceration, she maintained full color vision, indicating that while the corneal pathology affected her visual acuity, the retinal and optic nerve function remained intact. The examination further demonstrated no relative afferent pupillary defect (rAPD) and extraocular muscle (EOM) restriction in supraduction, infraduction, and abduction of the right eye. These findings culminated in the diagnosis of corneal ulceration secondary to idiopathic orbital inflammatory disease.

Her physical examination findings included significant bilateral orbital swelling (right greater than left), pronounced right lagophthalmos, and conjunctival injection without discharge. The examination noted severe right lagophthalmos with associated exposure keratopathy. Proptosis was objectively assessed using Hertel exophthalmometry, which revealed a 4 mm difference in proptosis OD (right eye greater than left eye).

She was admitted for inpatient management, starting on IV methylprednisolone 40 mg daily for six days and multiple eye treatments, including moxifloxacin drops, erythromycin ointment, and artificial tears. After excluding pre-septal cellulitis, orbital cellulitis, glaucoma, and neoplasms based on thorough diagnostic evaluations and consultations with an ophthalmologist and rheumatologist, it became clear that the best course of action was to treat the idiopathic inflammatory process with steroids alone. Although a repeat biopsy was considered, the decision was made to proceed with treatment based on the patient's clinical presentation, imaging findings, and prior biopsy results, which confirmed lacrimal gland inflammation consistent with nonspecific orbital inflammatory syndrome. The comprehensive lab workup, including autoimmune markers and thorough diagnostic evaluations further supported the diagnosis of IOID, thereby negating the need for an additional biopsy at this time. Rheumatology recommended a comprehensive lab work-up, which included immunoglobulin levels (ANA, ANCA, C3, and C4) for various autoimmune conditions. All results were within normal limits except for a positive ANCA and an elevated ANA titer of 1:320 (Table 2), which supported an autoimmune process. These results supported the diagnosis of an inflammatory process and helped exclude the other conditions mentioned above, reinforcing the decision to treat with corticosteroids.

**Table 2:** Immunology laboratory test results Note: Some tests like CD markers and complement levels may not have universal reference ranges and can vary based on the laboratory and specific assay methods. It's essential to interpret results within the context of the laboratory's provided reference ranges and clinical evaluation. Titers: represents the dilution level of a substance in the blood, often used for antibody measurements; Count/µL (counts per microliter): a measure of the number of cells or particles in one microliter of blood; mg/dL (milligrams per deciliter): a concentration measurement indicating the amount of substance in milligrams per deciliter of blood; U/mL (units per milliliter): a measure of the activity level of enzymes or other biochemicals in units per milliliter of blood; pg/mL (picograms per milliliter): a measure of very small quantities of substances (like hormones) in picograms per milliliter of blood; none detected: no presence of the specified antibodies or antigens found in the test; positive/negative: indicates whether antibodies, antigens, or other substances were detected as per the specified test.

Test name	Result	Units	Reference range
ANA antinuclear antibody titer	1:320 (positive)	Titers	Negative: <1:80
ANCA IFA	Positive	-	-
Anti-ds DNA antibody	None Detected	-	-
CD16+CD56 ABS	82	Count/µL	15 - 300
CD16+CD56 PCT	3	%	1 - 15
CD19 absolute	1,392	Count/µL	200 - 1,259
CD19 percent	49%	%	9 - 29%
CD3 absolute counts	1,354	Count/µL	1,072 - 3,890
CD3 percentage	47%	%	55 - 82%
CD4 absolute	648	Count/µL	562 - 2,692
CD4 percentage	23%	%	27 - 57%
CD4 to CD8 ratio	1.16	Ratio	.98 - 3.24
CD8 absolute	559	Count/µL	331 - 1,445
CD8 percentage	20%	%	14 - 34%
Complement 3	158	mg/dL	85 - 142
Complement 4	24	mg/dL	12 - 41
ENA screen	None detected	-	-
Fluorescent ANA	Positive	-	-
Hepatitis B surface antigen	Negative	-	-
Hepatitis C total Ab	Negative	-	-
HLAB27 genetic disease association	None detected	-	-
IFA titer	1:160 (positive)	Titers	Negative: <1:80
Myeloperoxidase AB	27.09	U/mL	0 - 20
Serine protease 3 AB	3	U/mL	0 - 20

On her sixth day in the hospital, she was stable for discharge and transitioned to oral prednisone 30 mg/day with a slow taper and gastric prophylaxis. Continued eye treatments and eye protection while sleeping were advised, with consideration for steroid-sparing agents or biologics on an outpatient basis. At the time of discharge, the corneal ulcer showed significant improvement, with reduced stromal thinning and decreased size. This was attributed to the intensive eye treatments, including moxifloxacin drops, erythromycin ointment, and artificial tears. The ulcer was healing, and ongoing monitoring was planned to ensure complete resolution.

## Discussion

This case involved an eight-year-old female presenting with an acute exacerbation of orbital symptoms over the course of one week. The rapid onset and progression of her symptoms, including worsening proptosis, pain, and redness, classified the presentation as acute. These symptoms raised concerns for several differential diagnoses, such as orbital cellulitis, pre-septal cellulitis, and glaucoma. The patient had experienced similar symptoms 18 months prior, which led to a diagnosis of bilateral lacrimal gland enlargement and a biopsy that identified nonspecific orbital inflammatory syndrome. This history indicates that the patient has been suffering from a chronic affliction, likely a recurrent or relapsing condition. The current episode was therefore treated as an acute exacerbation of IOID, guided by her prior diagnosis and the pattern of her symptoms.

Orbital and pre-septal cellulitis were initially considered due to their presentation with orbital swelling, erythema, and pain, which can mimic IOID. Pre-septal cellulitis is more common and less severe, defined as an infectious process of the subcutaneous eyelid tissues anterior to the orbital septum, without the involvement of the structures within the globe [8]. In contrast, orbital cellulitis is a potentially life-threatening condition caused by infection of the soft tissues posterior to the orbital septum. In pediatric patients, it is most commonly caused by *Streptococcus pneumoniae*, *Staphylococcus aureus*, or *Streptococcus pyogenes* [8]. These conditions were excluded due to the absence of systemic symptoms (fever, malaise) and lack of imaging showing abscesses or other infectious etiologies. Additionally, the patient’s lack of response to empirical antibiotics, which were initiated by the general pediatrics team upon admission, further supported the non-infectious nature of her condition.

Glaucoma was also considered due to the presentation of proptosis and eye redness, potentially caused by impaired intraocular fluid drainage or anatomical variants. Primary glaucoma, which arises without any identifiable underlying cause, was unlikely in this case. However, secondary glaucoma, which can develop as a result of another ocular or systemic condition, was considered, particularly due to the potential for orbital pathology to cause an acute rise in intraocular pressure. This consideration included associations with non-acquired ocular anomalies (such as Axenfeld-Rieger anomaly, Peters anomaly, and aniridia), as well as glaucoma associated with systemic diseases (such as Sturge-Weber syndrome and neurofibromatosis) [9]. Despite this, glaucoma was ruled out based on normal intraocular pressure through tonometry and the absence of optic nerve abnormalities [10]. The acute onset of symptoms and the response to corticosteroid therapy, along with these findings effectively excluded secondary glaucoma as a cause of her proptosis.

Neoplasms were initially considered malignant growths that could explain the mass effects observed. However, the absence of progressive symptoms and normal imaging findings, coupled with a lack of systemic markers typically associated with malignancy, such as elevated LDH and specific tumor markers like alpha-fetoprotein (AFP) and carcinoembryonic antigen (CEA), helped to exclude cancerous etiologies [11]. It’s important to note that these markers are generally used to monitor disease progression or response to treatment rather than to diagnose a neoplasm. In this case, the normal levels of these markers, along with the clinical and imaging findings, made a malignant process unlikely.

Therefore, our diagnostic approach involved a meticulous process of exclusion, as IOID is a diagnosis that can only be made after ruling out other potential causes. This process began with a thorough clinical evaluation, where conditions such as orbital cellulitis, pre-septal cellulitis, neoplasms, and glaucoma were considered. Orbital cellulitis and pre-septal cellulitis were initially suspected due to the patient’s symptoms of orbital swelling, erythema, and pain. However, the absence of systemic symptoms such as fever, a lack of purulent discharge, and negative blood cultures, along with imaging that did not show the typical signs of infection or abscess formation, made these diagnoses less likely. Neoplasms were ruled out through imaging studies that did not reveal any mass lesions or structural abnormalities consistent with malignancy. Additionally, a lack of progressive symptoms and normal levels of tumor markers supported the exclusion of neoplastic causes. Glaucoma, specifically secondary glaucoma, was considered due to the ocular symptoms; however, normal intraocular pressure measurements and the absence of optic nerve abnormalities on examination effectively excluded this possibility. The patient’s laboratory testing was also integral to the exclusion process. Negative autoimmune panels, including ANA and ANCA, along with normal thyroid function tests, helped rule out autoimmune causes. The patient’s lack of response to empirical antibiotics further supported the exclusion of infectious etiologies. After systematically ruling out these conditions, IOID emerged as the most likely diagnosis, particularly given the patient's clinical presentation of worsening right eye proptosis, pain with extraocular movements, and severe lagophthalmos leading to corneal ulceration, all features consistent with IOID [12]. This careful exclusion process, combined with the patient’s history and clinical findings, led to the determination that the current episode was an acute exacerbation of a chronic IOID condition. Inpatient management allowed for careful monitoring and adjustment of corticosteroid therapy, followed by a transition to oral prednisone with a gradual tapering schedule. Collaboration between ophthalmology and rheumatology ensured comprehensive care and consideration of effective long-term management strategies.

The patient’s positive response to corticosteroid therapy, without adverse systemic effects, indicated a favorable prognosis. Long-term follow-up will focus on monitoring disease progression and managing potential steroid-related side effects, such as dermatological issues, electrolyte abnormalities, hypertension, hyperglycemia, or pancreatitis [13]. If steroid therapy proves problematic, alternative treatments like monoclonal antibodies, such as rituximab [12], may be considered based on the patient's ongoing presentation.

In regards to continuity of care, given the chronic nature of IOID and the potential for recurrence, this patient will require regular follow-up visits. At first, monthly ophthalmologic assessments are recommended, gradually shifting to every three to six months based on the stability of her condition. These visits should include detailed eye examinations to monitor for signs of relapse, particularly proptosis, pain, or vision changes, and to assess for steroid-related complications such as ocular hypertension, cataracts, or further corneal damage. Systemic monitoring should include regular checks of blood pressure, blood glucose, and electrolytes to manage potential steroid side effects [14]. If relapses occur or if long-term steroid use leads to significant side effects, the introduction of steroid-sparing agents, such as methotrexate or rituximab, should be considered [12]. Educating the patient and family about the signs of relapse and the importance of medication adherence is essential for early detection and management. Therefore, vigilant follow-up is necessary to adjust treatment as needed and prevent complications.

## Conclusions

This case report offers several valuable insights into the diagnosis and management of IOID in a pediatric patient, a condition that is relatively rare in this age group. While the diagnostic process relied on a method of exclusion, this approach was far from routine. The systematic exclusion of other potentially more severe conditions, such as orbital cellulitis, neoplasms, and glaucoma, was particularly challenging due to the overlap of symptoms. This case highlights the necessity of a detailed and careful diagnostic process, which involves the integration of clinical findings, imaging studies, laboratory results, and the patient’s history.

This case is novel in several ways. Firstly, the acute exacerbation of a chronic IOID condition in a pediatric patient is not commonly reported in the literature, particularly with such a rapid progression of symptoms. The identification and management of this exacerbation add to the limited knowledge of how IOID can present and evolve in children. Secondly, the successful management of this case underscores the critical role of multidisciplinary collaboration, particularly between ophthalmology and rheumatology, which was pivotal in both the acute and long-term management of the disease. Moreover, the case underscores the importance of considering IOID in the differential diagnosis of pediatric patients presenting with similar ocular symptoms, especially when other more common and severe conditions have been ruled out. This highlights the need for awareness among clinicians about the potential for IOID to present with acute and severe symptoms in children, as it can be effectively managed with prompt and appropriate treatment.
